# Integrated ^18^F-T807 Tau PET, Structural MRI, and Plasma Tau in Tauopathy Neurodegenerative Disorders

**DOI:** 10.3389/fnagi.2021.646440

**Published:** 2021-03-29

**Authors:** Cheng-Hsuan Li, Ta-Fu Chen, Ming-Jang Chiu, Ruoh-Fang Yen, Ming-Chieh Shih, Chin-Hsien Lin

**Affiliations:** ^1^Department of Neurology, National Taiwan University Hospital, Taipei, Taiwan; ^2^Department of Neurology, National Taiwan University Biomedical Park Hospital, Hsinchu, Taiwan; ^3^Graduate Institute of Brain and Mind Sciences, College of Medicine, National Taiwan University, Taipei, Taiwan; ^4^Graduate Institute of Biomedical Engineering and Bioinformatics, National Taiwan University, Taipei, Taiwan; ^5^Graduate Institute of Psychology, National Taiwan University, Taipei, Taiwan; ^6^Department of Nuclear Medicine, National Taiwan University Hospital, Taipei, Taiwan; ^7^Institute of Epidemiology and Preventive Medicine, College of Public Health, National Taiwan University, Taipei, Taiwan

**Keywords:** tauopathies, Tau PET, biomarker, 18F-T807, progressive supranuclear palsy (PSP), corticobasal syndrome (CBS)

## Abstract

**Background and Objective:** Tau-specific positron emission topography (PET) imaging enables *in vivo* assessment of Alzheimer's disease (AD). We aimed to investigate its performance in combination with plasma tau levels in patients with non-AD tauopathy.

**Methods:** A total of 47 participants were enrolled, including 10 healthy controls, 16 with tauopathy parkinsonism syndromes (9 with corticobasal syndrome [CBS], 7 with progressive supranuclear palsy [PSP]), 9 with frontotemporal dementia (FTD), 4 with AD, and 8 with Parkinson's disease (PD). All participants underwent clinical assessments, ^18^F-T807 tau PET, brain MRI, and plasma tau assay.

**Results:** The global cortical standard uptake value ratio (SUVR) of ^18^F-T807 PET was comparable between PD and control (*p* = 0.088). The cortical SUVR was significantly higher in AD group (*p* = 0.002) but was modestly increased in PSP group compared to the PD group (*p* = 0.044), especially in parietal and pallidal regions. Asymmetric ^18^F-T807 uptake at the pallidum was noted in patients with CBS and FTD. Cortical tau tracer uptake was associated with increased plasma total tau level (*p* = 0.016), especially in frontal and parietal regions. Regional tracer uptake was correlated with cortical thinning in patients with CBS and PSP (CBS: *r* = −0.092, *p* = 0.025; PSP: *r* = −0.114, *p* = 0.015).

**Conclusions:** The ^18^F-T807 tau tracer uptake was only modestly increased in patients with PSP. Although the cortical tau tracer uptake correlated with regional cortical atrophy and plasma tau levels, a four-repeated tau-specific tracer is needed for future classifying tauopathy parkinsonism syndromes.

## Introduction

Misfolded tau protein deposits in neurons represent the neuropathological hallmark in many neurodegenerative disorders, including Alzheimer's disease (AD), frontotemporal dementia (FTD), progressive supranuclear palsy (PSP), corticobasal syndrome (CBS), argyrophilic grain disease, and Down syndrome (Murray et al., 2014; Wang and Edison, 2019). Normal tau is unfolded in neurons and has important physiological functions that stabilize microtubules and support axonal transport (Wang and Mandelkow, 2015). Tau is encoded by the microtubule-associated protein tau (*MAPT*) gene, and alternative splicing results in six tau isoforms in human brain, categorized into three-repeat (3R) or four-repeat (4R) tau according to the number of carboxy-terminal repeat domains (Wang and Mandelkow, 2015). AD is characterized by aggregates of mixed 3R and 4R tau in neurons, while non-AD tauopathy-related atypical parkinsonism, PSP and CBS, is characterized by neuronal 4R tau aggregates. Although PSP and CBS have common neuropathological forms of 4R tau inclusion, some features are distinct: PSP presents with tufted astrocytes in motor cortex, striatum, and midbrain; while CBS presents with astrocytic plaques in asymmetric atrophic cortices (Dickson, 1999).

Recently, the advent of tau-specific positron emission tomography (PET) has enabled *in vivo* detection of pathological tau aggregates in patients with AD (Ossenkoppele et al., 2016; Mattsson et al., 2017). Of all the tau PET tracers, ^18^F-T807 (also known as ^18^F-AV-1451, ^18^F-flortaucipir, or TAUVID) is the most widely applied in patients with neurodegenerative diseases and has been the first tau PET tracer approved by the U.S. Food and Drug Administration (Okamura et al., 2014a; Barthel, 2020; Fleisher et al., 2020). In patients with AD, ^18^F-T807 tracer retention is associated with disease severity and tracer bindings in inferior temporal, posterior cingulate, and lateral parietal regions parallel with the histopathological findings of tau-containing neurofibrillary tangles (Okamura et al., 2014b; Fleisher et al., 2020). However, the implications of ^18^F-T807 PET for patients with for non-AD tauopathy has yet to be confirmed. An alternative approach to assess tau pathology *in vivo* is measurement of biofluid levels of tau. Plasma levels of phosphorylated tau (p-tau181) were recently shown to be a surrogate blood-based biomarker of AD (Karikari et al., 2020). The current revised diagnostic guidelines for AD suggest that AD can be defined in living persons as a biological construct identifiable by changes in biomarkers that are indicative of the disease neuropathology independent of the manifestation of clinical symptoms; for example, the increased tau protein levels in cerebral spinal fluids (Jack et al., 2018). A blood-based tau assay has recently emerged because of blood's easy accessibility for repeated measurement (Barthélemy et al., 2020). However, studies examining the applications of either tau PET imaging or blood-based tau biomarkers in assisting the diagnosis of non-AD tauopathies are still few.

Here, we aimed to investigate a variety of non-AD tauopathies with an integrated approach combining ^18^F-T807 tau PET imaging, structural brain MRI, and plasma biomarkers including tau protein. We examined the differences in tau PET imaging and plasma biomarkers in AD and non-AD tauopathy compared with synucleinopathy disorder of Parkinson's disease (PD). We further explored the correlations between tau PET signal intensity and plasma tau level or structural imaging changes in cortical thickness.

## Materials and Methods

### Study Participants

All participants were recruited from the movement disorder or dementia special clinics of National Taiwan University Hospital (NTUH). A total of 47 participants were enrolled, including 10 neurologically normal controls, 16 with tauopathy parkinsonism syndromes (9 with CBS, 7 with PSP), 9 with FTD, 4 with AD, and 8 with PD. CBS was diagnosed according to the proposed criteria, exclusively the clinical phenotypes of probable corticobasal syndrome (Armstrong et al., 2013). Patients with PSP were diagnosed according to Movement Disorder Society Criteria (Höglinger et al., 2017); the clinical features of all patients met the diagnostic certainty of probable PSP and classified into the subtype of PSP-Richardson's syndrome. Patients with FTD, mainly the behavior variant of FTD or primary progressive aphasia with non-fluent/agrammatic or semantic variants, were recruited according to the diagnostic criteria (Gorno-Tempini et al., 2011; Rascovsky et al., 2011). AD was diagnosed according to the National Institute on Aging and Alzheimer's Association (NIA-AA) guideline (Mckhann et al., 2011). PD was diagnosed according to the United Kingdom PD Society Brain Bank clinical diagnostic criteria (Hughes et al., 1992). Patients were excluded if they presented with severe psychiatric symptoms or autonomic dysfunction within 1 year of parkinsonism symptom, history of stroke, vascular parkinsonism, neuroleptic-agent related parkinsonism, severe metabolic derangements, or were unable to cooperate with MRI or PET scans. Because patients with PSP, CBS, or FTD usually progress faster than patients with PD or AD, we only recruited patients that could be partially independent with modified Rankin scale ≤ 3 and can receive all the examinations safely in the current study. Healthy controls were neurologically normal participants recruited in the same institute. In addition to brain MRI, other neuroimaging studies were performed for assisting the clinical diagnosis in selected patients, including the Tc-99m TRODAT-1 dopamine transporter single photon emission tomography (SPECT) and ^18^F-FDG PET scans. To ensure the diagnostic accuracy, all participants underwent clinical follow-up in our clinics for at least 3 years. All participants provided informed consent before entering the study. The research protocols were approved by the Institutional Research Board Committee of NTUH.

### Clinical Assessments

All participants received a standard laboratory survey, parkinsonism motor symptoms, and cognitive function evaluations. The motor severity was evaluated using the subscale of the Unified Parkinson's Disease Rating Scale (UPDRS part III) (Goetz et al., 2008) for patients with parkinsonism features. Global cognitive functions were assessed using the total score on the Mini-Mental State Examination (MMSE) (Folstein et al., 1975), and all patients with AD or FTD received complete neuropsychological tests to support the clinical diagnosis.

### MRI Acquisition and Image Analysis

All participants underwent 1.5-T brain MRI scan during enrollment. High-resolution T1-weighted volumetric MRI were processed using Freesurfer software (version 6.0, http://surfer.nmr.mgh.harvard.edu) and its automatic multi-stage pipeline (recon-all command) for surface extraction, cortical parcellation, and cortical thickness estimation. The Desikan-Killiany atlas (i.e., lh.aparc.stats and rh.aparc.stats in Freesurfer output files) was adopted, and consisted of 34 predefined cortical regions of interest in each cerebral hemisphere (Desikan et al., 2006). Cortical thickness (CTh) was measured as the distance from the gray and white matter boundary to the corresponding pial surface. The estimated CTh data were expressed in millimeters (mm).

### Tau PET Acquisition, Image Processing, and Analysis

The tau tracer, ^18^F-T807, was prepared at the Cyclotron and Radiopharmaceutical Laboratory of the Department of Nuclear Medicine of NTUH as previously described (Huang et al., 2019). The ^18^F-T807 was synthesized in a FX_FN_ module (GE Healthcare, Milwaukee, WI). All participants underwent PET scans in a Biograph mCT PET/CT scanner (Siemens Medical Solutions; Malvern, PA, USA). PET imaging 3D acquisition was acquired for 20 min, starting at 80 min after intravenous bolus injection of 10 mCi ^18^F-T807 (Wooten et al., 2017; Heurling et al., 2019) by using 4-time frames with each of 5 min, and then the radioactivity was averaged using this 20-min acquisition protocol. PET data were reconstructed with ordered-set expectation maximization, corrected for attenuation, and then each frame was evaluated to verify adequate count statistics and absence of head motion. The reconstructed images had a matrix size of 400 × 400 × 148 and a voxel size of 0.68 × 0.68 × 1.5 mm^3^. PET data processing was performed using SPM12 (https://www.fil.ion.ucl.ac.uk/spm/) implemented in MATLAB 9.6 (MathWorks, Sherborn, MA). After segmentation of high-resolution T1-weighted MR images, the ^8^F-T807 PET data were co-registered on the corresponding MRI in naïve space. We used PETPVE12 (Gonzalez-Escamilla et al., 2017), a toolbox for SPM12, for partial-volume effect (PVE) correction of PET images by the geometric transfer matrix method. The standardized uptake value ratio (SUVR) was calculated using the cerebellar gray matter as the reference region in naïve space. The same Desikan-Killiany atlas mask was applied to each corrected PET image for region of interest (ROI)-based PET tracer SUVR calculation. Divided small cortical maps were merged into atlas-defined frontal, lateral temporal, parietal, and occipital regions (Desikan et al., 2006), and finally the global region was defined as all the cortical regions merged. PET tracer SUVR of atlas-defined putamen and pallidum were also calculated. The magnitude of hemispheric asymmetry of tau tracer uptake was evaluated by use of the asymmetry index (AI), defined as the absolute value of SUVR differences between the left and right side divided by the sum of SUVR between them [AI = | Left – Right |/(Left + Right) ^*^ 200]. A higher AI indicates more asymmetry between bilateral hemispheres in a specific region. Original ^18^F-T807 PET data, which were not corrected for partial-volume effect, were also processed and calculated for SUVR using the method described above.

### Measurement of Plasma Biomarkers

A total of 10 mL of venous blood was drawn from each participant and centrifuged (2,500 × g for 15 min) within 1 h after collection, and the samples were subsequently stored at −80°C until the test was performed. The plasma samples were analyzed using the Simoa platform (Quanterix, Lexington, MA) for measuring total tau, neurofilament light chain (NFL), glial fibrillary acidic protein (GFAP), and ubiquitin carboxyl-terminal hydrolase L1 (UCH-L1) (Neurology 4-Plex A assay kit). All samples were run in duplicates, and two internal quality-control plasma samples were run at the beginning and end of each run.

### Statistical Analysis

Continuous variables were summarized with means and standard deviations. Basic clinical characteristics of groups were compared using the Kruskal-Wallis test. For each region (global, frontal, lateral temporal, parietal, occipital, putamen, and pallidum), the SUVR values of the ^18^F-T807 tau PET scans were compared between groups using the Kruskal-Wallis test, with a *post hoc* Conover test comparing each disease group to PD, adjusted by Bonferroni correction. The asymmetry index was also compared between groups by use of the Kruskal-Wallis test. For the comparison of imaging findings or four plasma biomarkers between groups, analysis of covariance (ANCOVA) was used, with age and sex as covariates to control possible confounders. Bonferroni-corrected *p* values were adopted for *post hoc* pairwise comparisons between each group after ANCOVA. To investigate the relationship between plasma tau and tau PET tracer uptake in tauopathy-related disorders (CBS, PSP, FTD, and AD), we performed simple linear regression for the plasma tau level and non-corrected ^18^F-T807 SUVR value globally and in all cerebral regions. To account for possible confounding effects from age and sex, we also carried out a multiple linear regression model with age and sex entered as independent variables. We reported the slope coefficient (β1), standard error (SE), (partial) correlation coefficient (*r*), and *t* and *p* values for the slope coefficient for each model. Finally, to examine the relationship between cortical thickness and ^18^F-T807 tracer retention in given specific cerebral regions, Pearson correlation analysis was performed to compare the CTh (mm) with the SUVR value over the Desikan-Killiany atlas-defined cortical regions in each subject. The correlation coefficients from subjects within same disease group were meta-analyzed using the Hedges-Olkin method (Hedges Lv, 1985). We calculated the heterogeneity index *I*^2^ (Higgins and Thompson, 2002) and opted for the random-effects model if the heterogeneity index suggested substantial heterogeneity [*I*^2^ ≥ 50% (Higgins et al., 2020)]; otherwise, we used fixed-effect models. All statistical analyses were performed using MedCalc version 19.0.3 (MedCalc Software bvba, Ostend, Belgium). A *p* < 0.05 was considered statistically significant.

## Results

### Basic Clinical Characteristics

The demographic data of all 47 participants are summarized in Table 1. The participants' ages and disease duration were not statistically different between groups, although the duration of disease was numerically higher in patients with PD. The education level was higher in the PD than CBS group (*p* = 0.039). The MMSE score was lower in the AD group than in control (*p* < 0.001) or PD groups (*p* = 0.011). The scores were also lower in the FTD group than in control group (*p* = 0.008).

**Table 1:** Basic characteristics of the study participants.

	**Controls**	**PD**	**CBS**	**PSP**	**FTD**	**AD**	***p***
	**(*n* = 10)**	**(*n* = 8)**	**(*n* = 9)**	**(*n* = 7)**	**(*n* = 9)**	**(*n* = 4)**	
Sex (M/F)	2/8	2/6	2/7	3/4	4/5	3/1	
Age (year)	60.1 ± 10.4	66.4 ± 5.7	63.3 ± 13.2	70.0 ± 6.5	62.8 ± 6.9	70.0 ± 8.8	0.173
Disease duration (years)	-	8.1 ± 6.6	3.3 ± 1.0	4.7 ± 3.0	3.8 ± 1.8	5.5 ± 2.4	0.266
UPDRS III	-	18.1 ± 14.6	30.1 ± 10.0	39.4 ± 18.3	-	-	0.085
Education level (years)	14.3 ± 2.7	15.4 ± 3.2	9.3 ± 4.6	12.0 ± 3.4	12.5 ± 4.5	10.5 ± 5.3	0.041^a^
MMSE	29.2 ± 1.3	27.4 ± 4.3	24.7 ± 4.4	23.0 ± 4.4	21.0 ± 8.9	8.5 ± 6.6	<0.001^a^

*PD, Parkinson's disease; CBS, corticobasal syndrome; PSP, progressive supranuclear palsy; FTD, frontotemporal dementia; AD, Alzheimer's disease; UPDRS III, Unified Parkinson's Disease Rating Scale III; MMSE, Mini Mental State Examination*.

*Data presented as mean ± SD*.

a *Statistically significant difference between groups (p < 0.05) by Kruskal-Wallis test*.

### Tau PET Imaging in Parkinsonism and Dementia Syndromes

**Figure 1:**
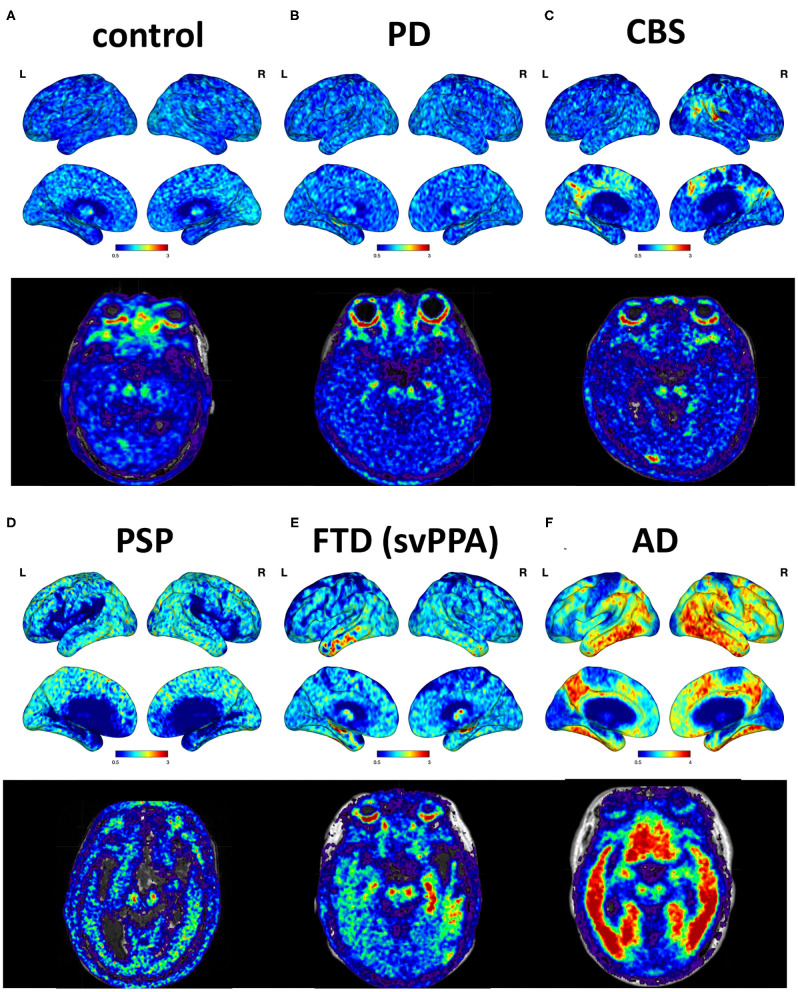
Cortical projections and midbrain axial views of representative subjects. **(A)** A 69-year-old male with normal neurological assessment. **(B)** A 57-year-old female with PD (UPDRS III: 28, MMSE: 29). **(C)** A 38-year-old male with possible CBS (MMSE: 29). **(D)** A 72-year-old male with PSP-Richardson syndrome (MMSE: 18). **(E)** A 76-year-old female with FTD, semantic variant PPA subtype (MMSE: 25). **(F)** A 64-year-old male with AD (MMSE: 16).

**Figure 2:**
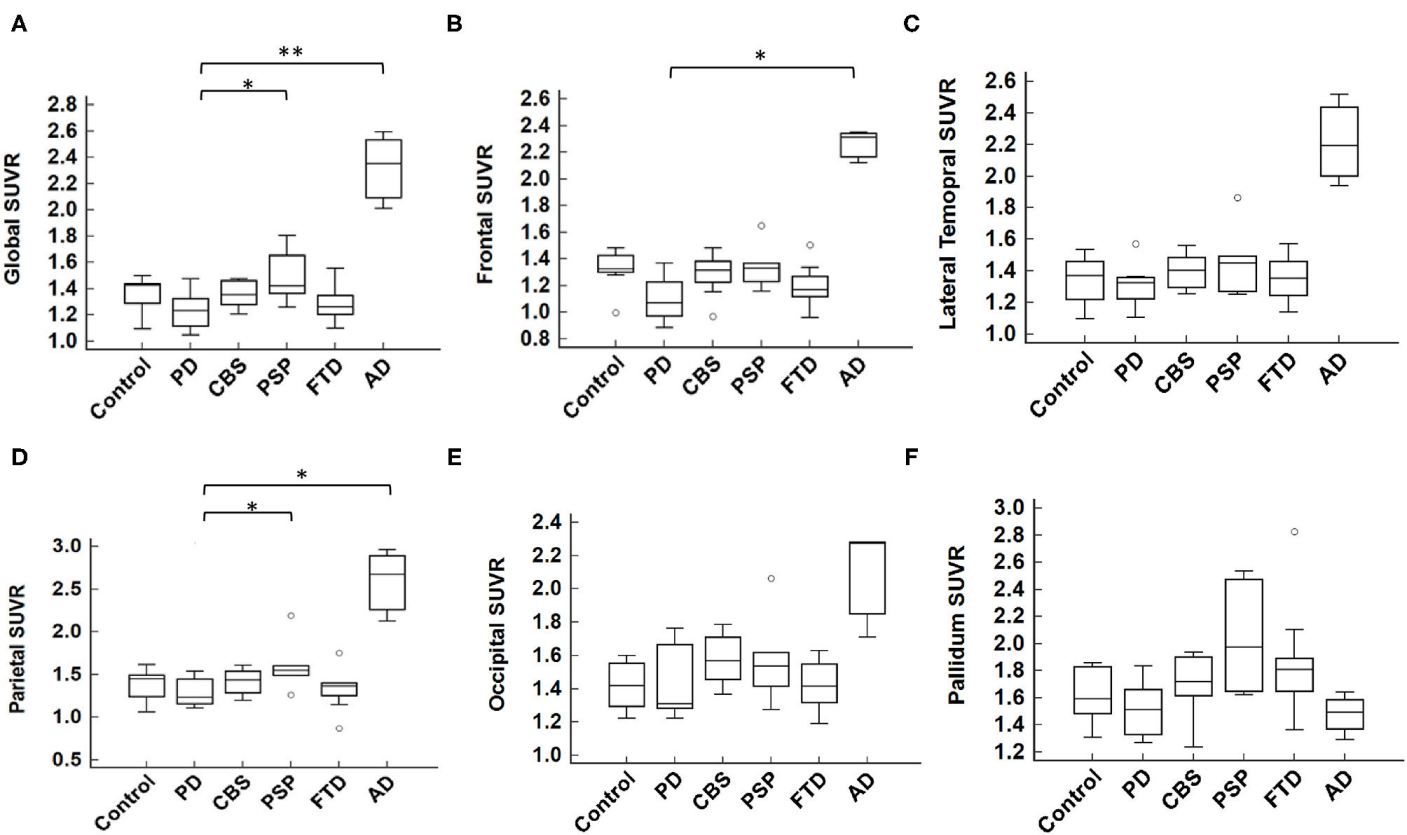
Mean ^18^F-T807 SUVR in global or regional cerebral cortex. **(A)** The global SUVR was significantly different between groups (*p* = 0.007, Kruskal-Wallis test). Comparing to the synucleinopathy disorder of PD, the global SUVR value of AD was statistically significantly higher than that of PD; while the PSP group had a modestly increased SUVR compared to the PD group (AD vs. PD, *p* = 0.002; PSP vs. PD, *p* = 0.044, by *post hoc* Conover test, with Bonferroni correction for multiple comparisons). In individual brain regions, including **(B)** frontal, **(C)** lateral temporal, **(D)** parietal, **(E)** occipital, and **(F)** pallidum, the SUVR was significantly higher in the frontal and parietal cortices in AD group and modestly higher in PSP group in the parietal cortex as compared to the PD group. *p < 0.05, **p < 0.01.

The cortical projection of tau tracer retention in representative subjects of each group is shown in Figure 1. The tau tracer retention in specific brain regions expressed as SUVR corrected for partial-volume effect, including globally and in the frontal, lateral temporal, parietal, and occipital regions, is shown in Table 2. There was no significant difference in either global or regional SUVR between PD and control groups (*p* = 0.088, Table 2, and Figure 2A). The global cortical SUVR was significantly higher in patients with AD (mean SUVR: 2.32) and was modestly increased in the PSP group (mean SUVR: 1.49) compared to controls or PD (mean SUVR: 1.24) (AD vs. PD, *p* = 0.002; PSP vs. PD, *p* = 0.044, Figure 2A). Regarding the individual regional tau retention in tauopathy-related disorders in comparison with the synuclienopathy of PD group, we observed a significantly higher SUVR in all individual regions in AD patients, especially in the frontal and parietal regions (*p* = 0.035 for the frontal region, *p* = 0.16 for the temporal region, *p* = 0.036 for the parietal region (Figures 2B–F and Table 2). Patients with PSP had a trend to have higher SUVR in parietal and pallidal regions than those in the PD group (parietal region: 1.61 ± 0.31 vs. 1.30 ± 0.17, *p* = 0.047; pallidal region: 2.05 ± 0.42 vs. 1.52 ± 0.20, *p* = 0.091) (Figures 2D,F and Table 2). Between-group comparisons of the asymmetry index showed differences over the pallidum region, where it was higher in the CBS, FTD, and AD groups than in the PD group, and higher in the CBS and AD groups than in the PSP group (all *p* < 0.05, Table 2). All disease groups showed similar SUVR in the pallidal regions, albeit the PSP group had a trend to have higher uptake than the PD or control group (Figure 2F). The ^18^F-T807 tracer uptake in specific brain regions as SUVR without partial-volume effect correction revealed similar results (Supplementary Table 1).

**Table 2:** Tau PET ^18^F-T807 SUVR and asymmetric index corrected for partial-volume effect in different disease groups.

	**Control**		**PD**		**CBS**		**PSP**		**FTD**		**AD**		
	**SUVR**	**AI (%)**	**SUVR**	**AI (%)**	**SUVR**	**AI (%)**	**SUVR**	**AI (%)**	**SUVR**	**AI (%)**	**SUVR**	**AI (%)**	***p***
Global	1.36 ± 0.12		1.24 ± 0.15		1.36 ± 0.10		1.49 ± 0.20^a^		1.29 ± 0.13		2.32 ± 0.29^b^		0.007
Frontal	1.32 ± 0.13	3.74 ± 3.45	1.11 ± 0.17	3.35 ± 2.68	1.29 ± 0.16	3.98 ± 2.11	1.35 ± 0.17	5.46 ± 2.95	1.20 ± 0.15	3.73 ± 2.51	2.26 ± 0.12^c^	7.75 ± 9.75	0.004
Lat Temporal	1.35 ± 0.14	5.26 ± 5.46	1.31 ± 0.15	4.75 ± 3.26	1.40 ± 0.11	4.75 ± 6.03	1.46 ± 0.22	4.23 ± 3.45	1.35 ± 0.15	4.92 ± 2.41	2.22 ± 0.29	3.87 ± 1.50	0.061
Parietal	1.38 ± 0.17	3.60 ± 3.01	1.30 ± 0.17	4.22 ± 3.15	1.42 ± 0.15	6.09 ± 10.2	1.61 ± 0.31^d^	7.25 ± 4.67	1.33 ± 0.23	6.70 ± 10.4	2.59 ± 0.43^e^	14.5 ± 11.2	0.013
Occipital	1.42 ± 0.14	5.34 ± 3.39	1.45 ± 0.22	4.85 ± 1.92	1.58 ± 0.15	3.70 ± 4.64	1.57 ± 0.27	12.4 ± 14.5	1.42 ± 0.14	5.32 ± 3.73	2.09 ± 0.33	15.2 ± 17.9	0.025
Putamen	1.48 ± 0.16	2.71 ± 1.98	1.40 ± 0.17	1.79 ± 1.51	1.49 ± 0.19	11.1 ± 14.0	1.71 ± 0.41	4.97 ± 6.36	1.71 ± 0.22^f^	6.20 ± 4.64	1.91 ± 0.11^g^	5.28 ± 4.05	0.007
Pallidum	1.62 ± 0.19	5.63 ± 5.11	1.52 ± 0.20	2.48 ± 1.17	1.71 ± 0.23	11.6 ± 12.6^h^, ^i^	2.05 ± 0.42	2.98 ± 1.99	1.86 ± 0.42	7.24 ± 6.59^j^	1.48 ± 0.15	7.06 ± 0.65^k^, ^l^	0.023

*PD, Parkinson's disease; CBS, corticobasal syndrome; PSP, progressive supranuclear palsy; FTD, frontotemporal dementia; AD, Alzheimer's disease; SUVR, standard uptake value ratio; AI, asymmetry index*.

*Data presented as mean ± SD. p: Kruskal-Wallis p value for SUVR between groups*.

SUVR values were compared between disease groups using the Kruskal-Wallis test, with a post-hoc Conover test comparing different disease groups to PD as reference group, adjusted by Bonferroni correction:

a PSP > PD (p = 0.044);

b AD > PD (p = 0.002);

c AD > PD (p = 0.035);

d PSP > PD (p = 0.047);

e AD > PD (p = 0.036);

f FTD > PD (p = 0.038);

g *AD > PD (p = 0.038)*.

AIs were compared between disease groups using Kruskal-Wallis test:

h CBS > PD;

i CBS > PSP;

j FTD > PD;

k AD > PD;

l AD > PSP (all p < 0.05)

### Comparisons of Plasma Biomarkers in Parkinsonism and Dementia Syndromes

The plasma levels of total tau, GFAP, NFL, and UCH-L1 in different disease groups are shown in Table 3. Among these biomarkers, GFAP was significantly correlated with the participant's age (*r* = 0.32, 95% CI 0.04–0.56, *p* = 0.03). After controlling potential confounders including age and sex, the plasma total tau level was not significantly different between groups (controls: 1.13 ± 0.66 pg/mL; PD: 2.00 ± 1.11 pg/mL; CBS: 1.66 ± 0.75 pg/mL; PSP: 1.99 ± 0.65 pg/mL; FTD: 1.78 ± 0.84 pg/mL; AD: 2.63 ± 0.47 pg/mL; by ANCOVA adjusted for age and sex), although the level was numerically higher in the AD dementia group. The plasma NFL level was significantly higher in the disease groups of PSP (68.8 ± 22.0 pg/mL, *p* = 0.003), CBS (58.6 ± 32.0 pg/mL, *p* = 0.003) and FTD (52.40 ± 21.80 pg/mL, *p* = 0.02) compared to those in the control group (9.82 ± 5.30 pg/mL). The plasma GFAP level was highest in the AD group (624.5 ± 92.8 pg/mL; all *p* < 0.002) followed by the PSP group (346.0 ± 65.5 pg/mL, *p* = 0.007). The UCH-L1 level was highest in the PSP group (49.0 ± 17.2 pg/mL, *p* = 0.002). These findings demonstrated AD group had the highest plasma markers of tau and GFAP, while PSP group had the highest level of NFL and UCH-L1 among all disease groups.

**Table 3:** Plasma biomarkers in individual disease groups.

	**Control**	**PD**	**CBS**	**PSP**	**FTD**	**AD**	***p***
	**(*n* = 10)**	**(*n* = 8)**	**(*n* = 9)**	**(*n* = 7)**	**(*n* = 9)**	**(*n* = 4)**	
Tau (pg/mL)	1.13 ± 0.66	2.00 ± 1.11	1.66 ± 0.75	1.99 ± 0.65	1.78 ± 0.84	2.63 ± 0.47	0.055
NfL (pg/mL)	9.82 ± 5.30	40.19 ± 33.62	58.55 ± 31.98^a^	68.81 ± 22.05^a^	52.40 ± 21.80^a^	50.14 ± 21.41	<0.001
GFAP (pg/mL)	124.1 ± 48.3	253.7 ± 168.9	210.7 ± 92.9	346.0 ± 65.5^b^	271.5 ± 125.1	624.5 ± 92.8^b^	<0.001
UCH-L1 (pg/mL)	16.3 ± 6.13	32.26 ± 20.15	32.64 ± 16.76	49.01 ± 17.16^c^	38.6 ± 9.63^c^	44.75 ± 14.33	0.001

*PD, Parkinson's disease; CBS, corticobasal syndrome; PSP, progressive supranuclear palsy; FTD, frontotemporal dementia; AD, Alzheimer's disease*.

*Data presented as mean ± SD. p: Kruskal-Wallis p value between groups*.

*The plasma biomarkers were compared between different groups using ANCOVA, with age and sex as covariates, and post-hoc pairwise comparisons using Bonferroni-corrected p values*.

a *NFL level: CBS > Control (p = 0.003); PSP > Control (p = 0.003); FTD > Control (p = 0.02)*.

b *GFAP level: PSP > Control (p = 0.007); AD > all other groups (p < 0.002)*.

c *UCH-L1 level: PSP > Control (p = 0.002); FTD > Control (p = 0.03)*.

### Regression Analysis of Tau PET Imaging Findings and Plasma Total Tau Level

**Figure 3:**
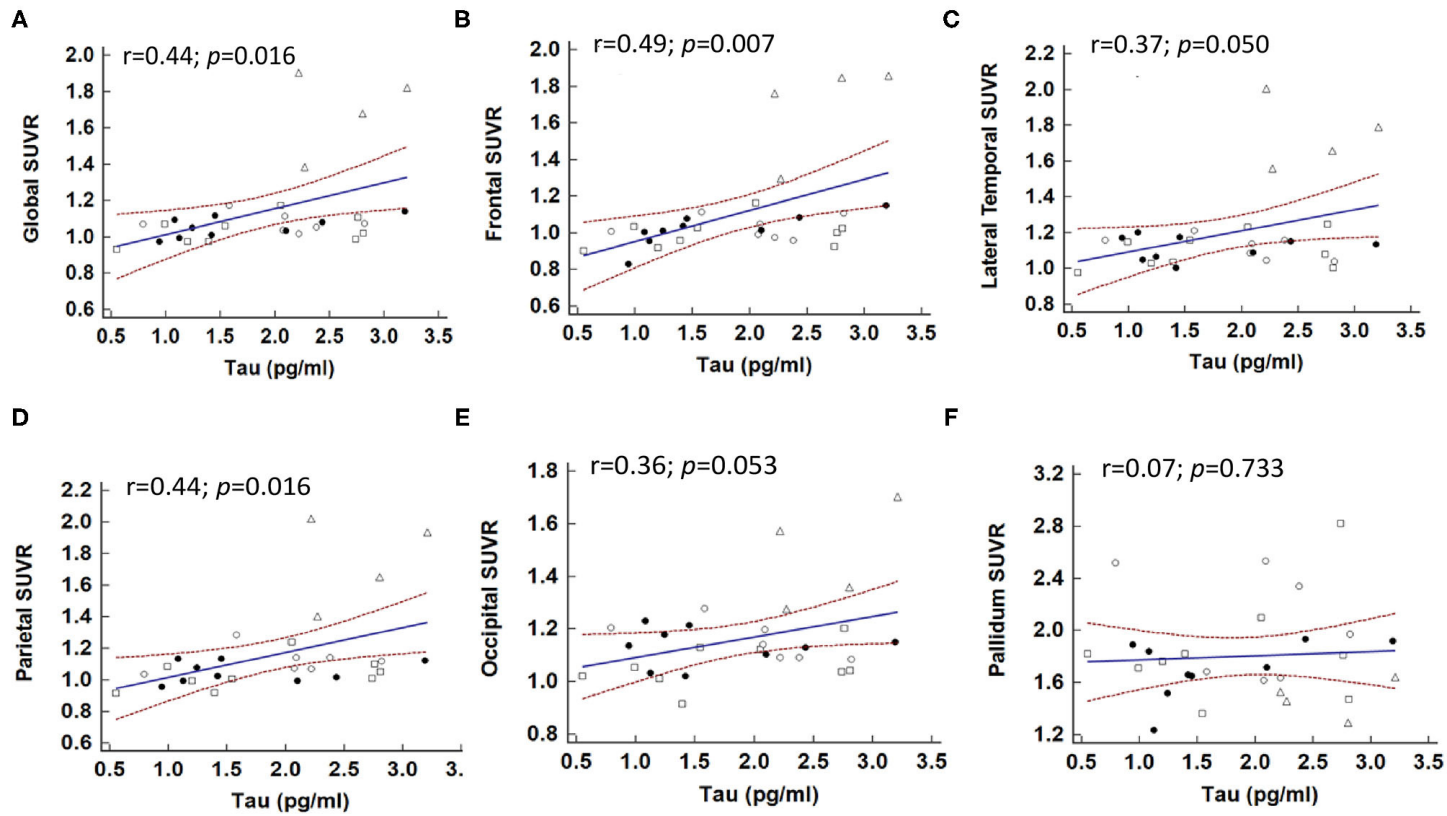
Correlations between plasma total tau level and brain regional SUVR in patients with tauopathies. The correlations between global **(A)** and individual brain regions, including **(B)** frontal, **(C)** lateral temporal, **(D)** parietal, **(E)** occipital, and **(F)** pallidum and plasma total tau level. Solid dot: CBS; open dot: PSP; square: FTD; triangle: AD.

For both AD and non-AD tauopathy disorders, the relations between plasma total tau level and tau PET imaging were examined using simple linear regression (Figure 3 and Supplementary Table 2). A higher SUVR value was significantly associated with higher total tau level globally (regression coefficient = 0.143, 95% CI: 0.029–0.256, *p* = 0.016), frontal (coefficient = 0.170, 95% CI: 0.051–0.289, *p* = 0.007), and parietal (coefficient = 0.158, 95% CI: 0.032–0.284, *p* = 0.016) regions (Figures 3A,B,D). There was also a trend for association between plasma tau and SUVR values in the lateral temporal (coefficient = 0.118, 95% CI: −0.0001–0.235, *p* = 0.0502) (Figure 3C) and occipital (coefficient = 0.077, 95% CI: −0.001–0.156, *p* = 0.053) (Figure 3E) regions. However, there was no obvious association between plasma tau and SUVR in the putamen or pallidum (Figure 3F). Furthermore, the correlation between regional cortical SUVR value and plasma tau level still remained globally (regression coefficient = 0.129, *p* = 0.044) and in the frontal (coefficient = 0.152, *p* = 0.024) and parietal (coefficient = 0.146, *p* = 0.04) regions after adjusting for age and sex in the multiple-regression model (Supplementary Table 2).

Considering the correlations maybe driven by the group of AD. We also performed the analyses in patients with non-AD tauopathy disorders only (i.e., CBS, PSP, and FTD) as shown in Supplementary Figure 2. There was no statistically significant association between plasma total tau level and global ^18^F-T807 SUVR value in simple linear regression analysis (*p* = 0.092). However, plasma total tau level was still associated with ^18^F-T807 SUVR in the frontal region (coefficient = 0.43, *p* = 0.033). This association was still statistically significant in multiple regression analysis (coefficient = 0.49, *p* = 0.023) after adjusting for age and sex.

### Correlation of Tau PET Tracer Retention and Regional Cortical Thickness

Since increased cortical tau deposition may suggest neuronal degeneration in tau tracer uptake regions, we next examined the correlation of regional tau PET tracer retention with cortical thickness. First, the relation of cortical thickness to tau PET SUVR value across all the 68 Desikan-Killiany atlas-defined cortical regions was examined in each subject via Pearson correlation, and subsequently all subjects from the same group were meta-analyzed (Table 4). Among the non-AD tauopathies, CBS and PSP, the regional CTh (in mm) was negatively correlated with the regional ^18^F-T807 SUVR value (CBS: correlation coefficient *r* = −0.092, 95% CI: −0.172–−0.012, *p* = 0.025; PSP: *r* = −0.114, 95% CI: −0.204–−0.023, *p* = 0.015) (Supplementary Figures 1A,B). In the AD group, the correlation was stronger, although it did not meet statistical significance (*r* = −0.209, 95% CI −0.407–0.008, *p* = 0.059) due to the sample size. The correlation between CTh and tau PET tracer retention was not observed in the PD or FTD group, as high estimated heterogeneity was observed (PD: Q = 14.86, *I*^2^ = 52.90%; FTD: Q = 18.95, *I*^2^ = 57.78%).

**Table 4:** Correlations between regional cortical thickness and T807 SUVR value in different disease groups.

**Group**	**Correlation coefficient**	**95% CI**	***p* value**	**Q**	***I*^**2**^%**	**P (heterogeneity)**
PD^a^	−0.027	−0.151–0.098	0.675	14.86	52.90	0.038
CBS	−0.092	−0.172 to −0.012	0.025^b^	7.21	0.00	0.514
PSP	−0.114	−0.204 to −0.023	0.015^b^	5.34	0.00	0.501
FTD^a^	−0.046	−0.169 to 0.079	0.474	18.95	57.78	0.015
AD^a^	−0.209	−0.407 to 0.008	0.059	9.83	69.49	0.020

*PD, Parkinson's disease; CBS, corticobasal syndrome; PSP, progressive supranuclear palsy; FTD, frontotemporal dementia; AD, Alzheimer's disease*.

*The correlation between regional cortical thickness and T807 SUVR value over the 68 brain regions defined in the Desikan-Killiany atlas were calculated for each subject and subsequently meta-analyzed using the Hedges-Olkin method*.

a *Random-effect models are used for the presence of heterogeneity*.

b *Statistically significant (p < 0.05)*.

Next, the correlation of cortical thickness and ^18^F-T807 tracer SUVR was performed over each atlas-defined cortical region among all patients in all disease groups (Supplementary Table 3). We found the most significantly correlated regions were the posterior cingulate (*r* = −0.623, *p* < 0.0001), the supramarginal (*r* = −0.565, *p* < 0.001), and banks of the superior temporal sulcus (bankssts, left: *r* = −0.504, *p* < 0.001; *r*ight: *r* = −0.516, *p* = 0.001).

## Discussion

Our results demonstrated the integrated findings of ^18^F-T807 tau PET images and their associations with plasma tau level and regional cortical atrophy detected by brain MRI in AD and non-AD tauopathy compared with synucleinopathy disorder of PD. AD patients had the highest ^18^F-T807 tracer retention in global cortical regions, while the PSP group had a trend of increased tau PET tracer signals globally and in parietal and pallidum regions. The patients with CBS and FTD had asymmetric tau PET tracer retention compared with the group of PD. While integrated with brain MRI and plasma markers, we found that ^18^F-T807 PET tracer retention in cortical regions, especially in the frontal and parietal regions, is correlated with plasma tau level. The increased regional ^18^F-T807 tracer retention also correlated with the regional cortical atrophy, mainly in posterior cingulate, the supramarginal and superior temporal sulcus, especially in patients with AD, PSP, and CBS. Our results suggested that ^18^F-T807 PET imaging is robust in AD group but only modestly applicable in the groups of PSP and CBS.

Consistent with previous studies and the recent approval by the U.S. FDA (Cho et al., 2016; Ossenkoppele et al., 2016; Mattsson et al., 2017), the increased pattern of ^18^F-T807 tracer was well-documented in our AD patients and has served as an imaging marker for detecting mixed 3R and 4R tau in paired helical filaments structures in AD patients. However, its role in imaging other tauopathies is less clear (Lowe et al., 2016; Hammes et al., 2018; Lyoo et al., 2018; Whitwell, 2018; Leuzy et al., 2019). One reason for this is its weaker binding affinity for straight-filament tau, which is more common in non-AD tauopathies (Marqui et al., 2015; Lowe et al., 2016). Another issue is its off-target binding to non-tau targets (Leuzy et al., 2019) such as monoamine oxidase B, which is abundant in basal ganglia, choroid plexus, and neuromelanin-rich substantia nigra. These deep nuclei are frequently affected by CBS and PSP pathology, which hamper studies of ^18^F-T807 tau PET images for use in diagnosing non-AD tauopathy (Leuzy et al., 2019).

However, recent case-series studies utilizing ^18^F-T807 PET imaging in patients with CBS, PSP, or FTD have emerged. One study in patients with CBS has shown that ^18^F-T807 uptake is correlated with pathological 4R tau burden in postmortem brain tissues (Josephs et al., 2016). Some studies have addressed the asymmetry of tau PET retention in frontal and parietal regions and in basal ganglia, where asymmetrical patterns were correlated with clinical presentations in patients with CBS and were distinct from the findings of AD patients (Cho et al., 2017a; Smith et al., 2017a; Niccolini et al., 2018). These observations are in line with our observation of a more asymmetric pattern in tau PET retention over the cortical regions and basal ganglia in patients with CBS compared with PD or PSP, suggesting that an asymmetric index of tau tracer uptake in both cortical and subcortical regions may be applied to patients with CBS.

For patients with PSP, although several ^18^F-T807 studies have suggested limited findings (Coakeley et al., 2017; Marqui et al., 2017), recent studies have revealed group differences between PSP and PD or healthy controls by ^18^F-T807 tau PET images. These mainly addressed the basal ganglia, midbrain, cerebellum, and other deep structures, followed by frontal or other cortical regions (Cho et al., 2017b; Passamonti et al., 2017; Smith et al., 2017b; Whitwell et al., 2017). In our study, the tau PET SUVR in patients with PSP was modestly increased in the cortical region (especially parietal lobe) and pallidum compared to the groups of PD or controls. We also observed a high variability of tracer signals in the basal ganglia (Table 2 and Figure 2F). This may be explained by the heterogeneity of both clinical findings and tau PET images in patients under the umbrella of PSP diagnosis. Our further findings of a correlation between regional ^18^F-T807 SUVR and the corresponding brain cortical thinning in patients with PSP are in accord with gray-matter volume loss in previous PSP studies (Sintini et al., 2019; Nicastro et al., 2020).

As for the FTD syndromes, different ^18^F-T807 retention patterns have been observed in specific subtypes of FTD, such as bvFTD and primary progressive aphasia (Tsai et al., 2019). In our study, patients with the semantic variant of primary progressive aphasia, which is mainly predicted to have TDP-43 pathology, also showed bilateral but asymmetric anterior temporal binding of ^18^F-T807 tracer (Figure 1). Whether it suggests off-target binding to TDP-43 protein or true binding to tau deposition is unknown. In a small autoradiographic study enrolling patients with primary progressive aphasia, the results did not support off-target binding to TDP-43 (Schaeverbeke et al., 2020). In regard to both the clinical and neuropathological heterogeneity of FTD, a further large-cohort study to confirm a role for the clinical application of ^18^F-T807 in various subtypes of FTD is needed.

Our study demonstrates that, in tauopathy-related diseases, plasma tau level is associated with cortical tau tracer retention, especially in the frontal and parietal lobes, but not associated with the magnitude of tau retention in basal ganglia. The finding of an association between plasma tau level and cortical tau PET retention remained after adjusting for age and sex. The absence of association in the basal ganglia might be explained by off-target binding effects. This supports the concept that combined plasma tau measurement and *in vivo* tau PET imaging could be applied in patients with suspected tauopathy disorders. Currently, academic research on blood-based biomarkers is still largely focused on AD. Correlations of plasma tau or, more specifically, phosphorylated tau, have been found in patients with AD (Mielke et al., 2018; Thijssen et al., 2020), but the results of attempts to differentiate atypical parkinsonism syndromes (CBS and PSP) from healthy controls are still conflicting (Lin et al., 2018; Thijssen et al., 2020). Likewise, our team previously reported that plasma p-tau181 level alone could not clearly differentiate CBS and PSP from PD (Lin et al., 2018). The combination of other phosphorylated forms of tau proteins in the plasma and molecular tau PET imaging needs further large-cohort investigation. Our study also revealed that plasma NfL levels were significantly elevated in all disease groups compared to controls. The expression of NfL is mainly in the myelinated axons and the concentration of NfL in either CSF or plasma would be increased in the conditions of neurodegeneration, including PD, AD, multiple sclerosis, and amyotrophic lateral sclerosis (Weston et al., 2017; Lin et al., 2019). However, although plasma NfL level is a sensitive marker for neurodegenerative disorders, it lacks disease specificity but can reflect the progression of the disease depending on the severity of neuronal injury (Weston et al., 2017; Lin et al., 2019).

In addition, recent evidence has shown that neuroinflammation is critical to the pathogenesis of neurodegenerative disorders, including tauopathy (Ransohoff, 2016; Laurent et al., 2018). Plasma levels of pro-inflammatory cytokines, including IL-1b, IL-2, and TNF-a (Park et al., 2020), and PET scans using mitochondrial 18 kDa translocator protein (TSPO, also known as PK-11195), which is expressed by activated microglia, have been shown to be alternative neuroinflammatory markers for the progression of neurodegenerative disorders (Best et al., 2019). Combing these neuroinflammatory markers may provide a more complete framework for characterizing individual neurodegenerative diseases in the future.

The main strength of our study is the inclusion of a variety of tauopathies, which were examined and analyzed by tau PET imaging, structural MRI, and blood-based biomarkers under the same protocol. To our knowledge, there are no prior published data from the simultaneous evaluation of tau PET and plasma tau levels in non-AD tauopathies. Through our study, we found that ^18^F-T807 PET imaging alone could not well-differentiate non-AD tauopathy from PD or controls. However, the correlations of the retention of ^18^F-T807 signal with cortical atrophy and plasma tau level may reflect its role as a surrogate marker for neurodegeneration. This study has some limitations. First, although we included patients with different tauopathy to examine whether ^18^F-T807 PET imaging could be used to differentiate tauopathy-parkinsonism disorders, ex: PSP and CBS, from synucleinopathy disorder of PD, the number of each group is limited. In this study, patients with AD were enrolled as a positive control, because U.S. FDA has recently approved the ^18^F-T807 tracer is an imaging marker for AD patients (Cho et al., 2016; Ossenkoppele et al., 2016; Mattsson et al., 2017). Therefore, we did not enroll large number of AD patients in this study. A future large cohort study enrolling more patients with both AD and non-AD tauopathy with different parkinsonism disorders is needed to confirm our findings. Second, ^18^F-T807 PET imaging still performs better in the diagnosis of AD, due to its higher binding affinity to mixed 3R and 4R isoforms in paired-helical filament tau aggregates and its off-target binding in basal ganglia. Newer generation tau tracers that are more 4R tau-specific with less off-target binding are warranted for assisting the pre-mortem diagnosis of non-AD tauopathies. Third, we do not have data for plasma phosphorylated tau, especially p-tau181, which might be better correlated with tau PET signals than total forms of tau. Blood phosphorylated tau, especially p-tau181, has been shown to provide good diagnostic performance in differentiating AD from other diseases. However, its role for differentiating non-AD tauopathy parkinsonism syndrome is still unclear. Our previous study has showed that plasma p-tau181 alone could not differentiate CBS or PSP from PD (Lin et al., 2018). In another larger cohort study, plasma p-tau181 also failed to differentiate non-AD tauopathy from health controls (Thijssen et al., 2020). Future studies examining the specificity of other phosphorylated forms of tau for assisting the diagnosis of non-AD tauopathy parkinsonism are needed. Third, the clinical diagnosis was not confirmed by neuropathology, and the lack of amyloid-β biomarkers might cause misclassification of underlying pathological diagnosis. However, the final diagnosis was based on thorough clinical examination, assisted neuroimaging studies, clinical follow-up, and complete neuropsychological tests, and was done according to the updated international diagnostic consensus. Last, although there are various disease groups included, more patients are needed in each group to make more statistical inference.

In conclusion, our findings suggest that ^18^F-T807 retention is robustly increased in AD but is not significantly increased in other non-AD tauopathies. Although the cortical tau uptake correlated with regional cortical atrophy and plasma tau levels in these tauopathies, a four-repeated tau-specific tracer is needed for classifying non-AD tauopathy parkinsonism syndromes in the future.

## Data Availability Statement

The original contributions presented in the study are included in the article/Supplementary Material, further inquiries can be directed to the corresponding author/s.

## Ethics Statement

The studies involving human participants were reviewed and approved by Institutional Research Board Committee of National Taiwan University Hospital. The patients/participants provided their written informed consent to participate in this study.

## Author Contributions

C-HLi and C-HLin: study concept and design. C-HLi, T-FC, M-JC, R-FY, M-CS, and C-HLin: acquisition of data. C-HLi, R-FY, M-CS, and C-HLin: analysis and interpretation of data. C-HLi: drafting the manuscript. C-HLi and C-HLin: critical revision of the manuscript for important intellectual content. C-HLi and M-CS: statistical analysis. C-HLin: study supervision. All authors contributed to the article and approved the submitted version.

## Conflict of Interest

The authors declare that the research was conducted in the absence of any commercial or financial relationships that could be construed as a potential conflict of interest.

## References

[B1] Armstrong M. J. Litvan I. Lang A. E. Bak T. H. Bhatia K. P. Borroni B.. (2013). Criteria for the diagnosis of corticobasal degeneration. Neurology 80, 496–503. 10.1212/WNL.0b013e31827f0fd1 23359374 PMC3590050

[B2] Barthel H. (2020). First tau PET tracer approved: toward accurate in vivo diagnosis of alzheimer disease. J. Nucl. Med. 61, 1409–1410. 10.2967/jnumed.120.252411 33004646

[B3] Barthélemy N. R. Horie K. Sato C. Bateman R. J. (2020). Blood plasma phosphorylated-tau isoforms track CNS change in Alzheimer's disease. J. Exp. Med. 217:e20200861. 10.1084/jem.20200861 32725127 PMC7596823

[B4] Best L. Ghadery C. Pavese N. Tai Y. F. Strafella A. P. (2019). New and old TSPO PET radioligands for imaging brain microglial activation in neurodegenerative disease. Curr. Neurol. Neurosci. Rep. 19:24. 10.1007/s11910-019-0934-y 30941587

[B5] Cho H. Baek M. S. Choi J. Y. Lee S. H. Kim J. S. Ryu Y. H.. (2017a). (18)F-AV-1451 binds to motor-related subcortical gray and white matter in corticobasal syndrome. Neurology 89, 1170–1178. 10.1212/WNL.0000000000004364 28814462

[B6] Cho H. Choi J. Y. Hwang M. S. Kim Y. J. Lee H. M. Lee H. S.. (2016). *In vivo* cortical spreading pattern of tau and amyloid in the Alzheimer disease spectrum. Annals Neurol. 80, 247–258. 10.1002/ana.24711 27323247

[B7] Cho H. Choi J. Y. Hwang M. S. Lee S. H. Ryu Y. H. Lee M. S.. (2017b). Subcortical (18) F-AV-1451 binding patterns in progressive supranuclear palsy. Mov. Disord. 32, 134–140. 10.1002/mds.26844 27813160

[B8] Coakeley S. Cho S. S. Koshimori Y. Rusjan P. Harris M. Ghadery C.. (2017). Positron emission tomography imaging of tau pathology in progressive supranuclear palsy. J. Cereb. Blood Flow Metab. 37, 3150–3160. 10.1177/0271678X16683695 28155586 PMC5584690

[B9] Desikan R. S. Segonne F. Fischl B. Quinn B. T. Dickerson B. C. Blacker D.. (2006). An automated labeling system for subdividing the human cerebral cortex on MRI scans into gyral based regions of interest. Neuroimage 31, 968–980. 10.1016/j.neuroimage.2006.01.021 16530430

[B10] Dickson D. W. (1999). Neuropathologic differentiation of progressive supranuclear palsy and corticobasal degeneration. J. Neurol. 246(Suppl 2), Ii6–15. 10.1007/BF03161076 10525997

[B11] Fleisher A. S. Pontecorvo M. J. Devous M. D. Sr. Lu M. Arora A. K. Truocchio S. P.. (2020). Positron emission tomography imaging with [18F]flortaucipir and postmortem assessment of alzheimer disease neuropathologic changes. JAMA Neurol. 77, 829–839. 10.1001/jamaneurol.2020.0528 32338734 PMC7186920

[B12] Folstein M. F. Folstein S. E. Mchugh P. R. (1975). “Mini-mental state”. A practical method for grading the cognitive state of patients for the clinician. J. Psychiatr. Res. 12, 189–198. 10.1016/0022-3956(75)90026-6 1202204

[B13] Goetz C. G. Tilley B. C. Shaftman S. R. Stebbins G. T. Fahn S. Martinez-Martin P.. (2008). Movement disorder society-sponsored revision of the unified parkinson's disease rating scale (MDS-UPDRS): scale presentation and clinimetric testing results. Mov. Disord. 23, 2129–2170. 10.1002/mds.22340 19025984

[B14] Gonzalez-Escamilla G. Lange C. Teipel S. Buchert R. Grothe M. J. (2017). PETPVE12: an SPM toolbox for partial volume effects correction in brain PET - application to amyloid imaging with AV45-PET. Neuroimage 147, 669–677. 10.1016/j.neuroimage.2016.12.077 28039094

[B15] Gorno-Tempini M. L. Hillis A. E. Weintraub S. Kertesz A. Mendez M. Cappa S. F.. (2011). Classification of primary progressive aphasia and its variants. Neurology 76, 1006–1014. 10.1212/WNL.0b013e31821103e6 21325651 PMC3059138

[B16] Hammes J. Drzezga A. Van Eimeren T. (2018). The role of tau imaging in parkinsonian disorders. Curr. Neurol. Neurosci. Rep. 18:86. 10.1007/s11910-018-0898-3 30293094

[B17] Hedges Lv O. I. (1985). Statistical Methods for Meta-analysis. London: Academic Press.

[B18] Heurling K. Smith R. Strandberg O. T. Schain M. Ohlsson T. Hansson O.. (2019). Regional times to equilibria and their impact on semi-quantification of [(18)F]AV-1451 uptake. J. Cereb. Blood Flow Metab. 39, 2223–2232. 10.1177/0271678X18791430 30073880 PMC6827127

[B19] Higgins Jpt T.J. Chandler J. Cumpston M. Li T. Page M.j Welch V. (Editors) (2020). Cochrane Handbook for Systematic Reviews of Interventions version 6.1 (updated September 2020). Cochrane. 10.1002/9781119536604

[B20] Higgins J. P. Thompson S. G. (2002). Quantifying heterogeneity in a meta-analysis. Stat. Med. 21, 1539–1558. 10.1002/sim.1186 12111919

[B21] Höglinger G. U. Respondek G. Stamelou M. Kurz C. Josephs K. A. Lang A. E.. (2017). Clinical diagnosis of progressive supranuclear palsy: the movement disorder society criteria. Mov. Disord. 32, 853–864. 10.1002/mds.26987 28467028 PMC5516529

[B22] Huang Y. Y. Chiu M. J. Yen R. F. Tsai C. L. Hsieh H. Y. Chiu C. H.. (2019). An one-pot two-step automated synthesis of [18F]T807 injection, its biodistribution in mice and monkeys, and a preliminary study in humans. PLoS ONE 14:e0217384. 10.1371/journal.pone.0217384 31260447 PMC6602418

[B23] Hughes A. J. Daniel S. E. Kilford L. Lees A. J. (1992). Accuracy of clinical diagnosis of idiopathic Parkinson's disease: a clinico-pathological study of 100 cases. J. Neurol. Neurosurg. Psychiatr. 55, 181–184. 10.1136/jnnp.55.3.181 1564476 PMC1014720

[B24] Jack C. R. Jr. Bennett D. A. Blennow K. Carrillo M. C. Dunn B. Haeberlein S. B.. (2018). NIA-AA research framework: toward a biological definition of Alzheimer's disease. Alzheimers Dement 14, 535–562. 10.1016/j.jalz.2018.02.018 29653606 PMC5958625

[B25] Josephs K. A. Whitwell J. L. Tacik P. Duffy J. R. Senjem M. L. Tosakulwong N.. (2016). [18F]AV-1451 tau-PET uptake does correlate with quantitatively measured 4R-tau burden in autopsy-confirmed corticobasal degeneration. Acta Neuropathol. 132, 931–933. 10.1007/s00401-016-1618-1 27645292 PMC5107140

[B26] Karikari T. K. Pascoal T. A. Ashton N. J. Janelidze S. Benedet A. L. Rodriguez J. L.. (2020). Blood phosphorylated tau 181 as a biomarker for Alzheimer's disease: a diagnostic performance and prediction modelling study using data from four prospective cohorts. Lancet Neurol. 19, 422–433. 10.1016/S1474-4422(20)30071-5 32333900

[B27] Laurent C. Buée L. Blum D. (2018). Tau and neuroinflammation: what impact for Alzheimer's disease and tauopathies? Biomed. J. 41, 21–33. 10.1016/j.bj.2018.01.003 29673549 PMC6138617

[B28] Leuzy A. Chiotis K. Lemoine L. Gillberg P. G. Almkvist O. Rodriguez-Vieitez E.. (2019). Tau PET imaging in neurodegenerative tauopathies-still a challenge. Mol. Psychiatr. 24:1112–34. 10.1038/s41380-018-0342-8 30635637 PMC6756230

[B29] Lin C. H. Li C. H. Yang K. C. Lin F. J. Wu C. C. Chieh J. J.. (2019). Blood NfL: a biomarker for disease severity and progression in Parkinson disease. Neurology 93:e1104-11. 10.1212/WNL.0000000000008088 31420461

[B30] Lin C. H. Yang S. Y. Horng H. E. Yang C. C. Chieh J. J. Chen H. H.. (2018). Plasma biomarkers differentiate parkinson's disease from atypical parkinsonism syndromes. Front Aging Neurosci. 10:123. 10.3389/fnagi.2018.00123 29755341 PMC5934438

[B31] Lowe V. J. Curran G. Fang P. Liesinger A. M. Josephs K. A. Parisi J. E.. (2016). An autoradiographic evaluation of AV-1451 Tau PET in dementia. Acta Neuropathol. Commun. 4:58. 10.1186/s40478-016-0315-6 27296779 PMC4906968

[B32] Lyoo C. H. Cho H. Choi J. Y. Ryu Y. H. Lee M. S. (2018). Tau positron emission tomography imaging in degenerative parkinsonisms. J. Mov. Disord. 11, 1–12. 10.14802/jmd.17071 29381890 PMC5790630

[B33] Marqui,é M. Normandin M. D. Meltzer A. C. Siao Tick Chong M. Andrea N. V. Antón-Fernández A.. (2017). Pathological correlations of [F-18]-AV-1451 imaging in non-alzheimer tauopathies. Annals Neurol. 81, 117–128. 10.1002/ana.24844 PMC5319193 27997036

[B34] Marqui,é M. Normandin M. D. Vanderburg C. R. Costantino I. M. Bien E. A. Rycyna L. G.. (2015). Validating novel tau positron emission tomography tracer [F-18]-AV-1451 (T807) on postmortem brain tissue. Ann. Neurol. 78, 787–800. 10.1002/ana.24517 26344059 PMC4900162

[B35] Mattsson N. Schöll M. Strandberg O. Smith R. Palmqvist S. Insel P. S.. (2017). 18F-AV-1451 and CSF T-tau and P-tau as biomarkers in Alzheimer's disease. EMBO Mol. Med. 9, 1212–1223. 10.15252/emmm.201707809 28743782 PMC5582410

[B36] Mckhann G. M. Knopman D. S. Chertkow H. Hyman B. T. Jack C. R. Jr. Kawas C. H.. (2011). The diagnosis of dementia due to Alzheimer's disease: recommendations from the National Institute on Aging-Alzheimer's Association workgroups on diagnostic guidelines for Alzheimer's disease. Alzheimers Dement. 7, 263–269. 10.1016/j.jalz.2011.03.005 21514250 PMC3312024

[B37] Mielke M. M. Hagen C. E. Xu J. Chai X. Vemuri P. Lowe V. J.. (2018). Plasma phospho-tau181 increases with Alzheimer's disease clinical severity and is associated with tau- and amyloid-positron emission tomography. Alzheimers Dement 14, 989–997. 10.1016/j.jalz.2018.02.013 29626426 PMC6097897

[B38] Murray M. E. Kouri N. Lin W. L. Jack C. R. Jr. Dickson D. W. Vemuri P. (2014). Clinicopathologic assessment and imaging of tauopathies in neurodegenerative dementias. Alzheimers Res. Ther. 6:1. 10.1186/alzrt231 24382028 PMC3978456

[B39] Nicastro N. Rodriguez P. V. Malpetti M. Bevan-Jones W. R. Simon Jones P. Passamonti L.. (2020). (18)F-AV1451 PET imaging and multimodal MRI changes in progressive supranuclear palsy. J. Neurol. 267, 341–349. 10.1007/s00415-019-09566-9 31641878 PMC6989441

[B40] Niccolini F. Wilson H. Hirschbichler S. Yousaf T. Pagano G. Whittington A.. (2018). Disease-related patterns of *in vivo* pathology in Corticobasal syndrome. Eur. J. Nucl. Med. Mol. Imaging 45, 2413–2425. 10.1007/s00259-018-4104-2 30090966 PMC6208819

[B41] Okamura N. Furumoto S. Fodero-Tavoletti M. T. Mulligan R. S. Harada R. Yates P.. (2014a). Non-invasive assessment of Alzheimer's disease neurofibrillary pathology using 18F-THK5105 PET. Brain 137, 1762–1771. 10.1093/brain/awu064 24681664

[B42] Okamura N. Harada R. Furumoto S. Arai H. Yanai K. Kudo Y. (2014b). Tau PET imaging in Alzheimer's disease. Curr. Neurol. Neurosci. Rep. 14:500. 10.1007/s11910-014-0500-6 25239654

[B43] Ossenkoppele R. Schonhaut D. R. Scholl M. Lockhart S. N. Ayakta N. Baker S. L.. (2016). Tau PET patterns mirror clinical and neuroanatomical variability in Alzheimer's disease. Brain 139, 1551–1567. 10.1093/brain/aww027 26962052 PMC5006248

[B44] Park J. C. Han S. H. Mook-Jung I. (2020). Peripheral inflammatory biomarkers in Alzheimer's disease: a brief review. BMB Rep. 53, 10–19. 10.5483/BMBRep.2020.53.1.309 31865964 PMC6999828

[B45] Passamonti L. Vázquez Rodríguez P. Hong Y. T. Allinson K. S. Williamson D. Borchert R. J.. (2017). 18F-AV-1451 positron emission tomography in Alzheimer's disease and progressive supranuclear palsy. Brain 140, 781–791. 10.1093/brain/aww340 28122879 PMC5382948

[B46] Ransohoff R. M. (2016). How neuroinflammation contributes to neurodegeneration. Science 353, 777–783. 10.1126/science.aag2590 27540165

[B47] Rascovsky K. Hodges J. R. Knopman D. Mendez M. F. Kramer J. H. Neuhaus J.. (2011). Sensitivity of revised diagnostic criteria for the behavioural variant of frontotemporal dementia. Brain 134, 2456–2477. 10.1093/brain/awr179 21810890 PMC3170532

[B48] Schaeverbeke J. Celen S. Cornelis J. Ronisz A. Serdons K. Van Laere K.. (2020). Binding of [(18)F]AV1451 in post mortem brain slices of semantic variant primary progressive aphasia patients. Eur. J. Nucl. Med. Mol. Imaging 47, 1949–1960. 10.1007/s00259-019-04631-x 31848674 PMC7300115

[B49] Sintini I. Schwarz C. G. Senjem M. L. Reid R. I. Botha H. Ali F.. (2019). Multimodal neuroimaging relationships in progressive supranuclear palsy. Parkinsonism Related Disord. 66, 56–61. 10.1016/j.parkreldis.2019.07.001 31279635 PMC6779505

[B50] Smith R. Schain M. Nilsson C. Strandberg O. Olsson T. Hägerström D.. (2017b). Increased basal ganglia binding of (18) F-AV-1451 in patients with progressive supranuclear palsy. Mov. Disord. 32, 108–114. 10.1002/mds.26813 27709757 PMC6204612

[B51] Smith R. Schöll M. Widner H. Van Westen D. Svenningsson P. Hägerström D.. (2017a). *In vivo* retention of (18)F-AV-1451 in corticobasal syndrome. Neurology 89, 845–853. 10.1212/WNL.0000000000004264 28754841 PMC5580862

[B52] Thijssen E. H. La Joie R. Wolf A. Strom A. Wang P. Iaccarino L.. (2020). Diagnostic value of plasma phosphorylated tau181 in Alzheimer's disease and frontotemporal lobar degeneration. Nat. Med. 26, 387–397. 10.1038/s41591-020-0762-2 32123386 PMC7101073

[B53] Tsai R. M. Bejanin A. Lesman-Segev O. Lajoie R. Visani A. Bourakova V.. (2019). (18)F-flortaucipir (AV-1451) tau PET in frontotemporal dementia syndromes. Alzheimers Res. Ther. 11:13. 10.1186/s13195-019-0470-7 30704514 PMC6357510

[B54] Wang Y. Mandelkow E. (2015). Tau in physiology and pathology. Nat. Rev. Neurosci. 17, 22–35. 10.1038/nrn.2015.1 26631930

[B55] Wang Y. T. Edison P. (2019). Tau imaging in neurodegenerative diseases using positron emission tomography. Curr. Neurol. Neurosci. Rep. 19:45. 10.1007/s11910-019-0962-7 31172290 PMC6554240

[B56] Weston P. S. J. Poole T. Ryan N. S. Nair A. Liang Y. Macpherson K.. (2017). Serum neurofilament light in familial Alzheimer disease: a marker of early neurodegeneration. Neurology 89, 2167–2175. 10.1212/WNL.0000000000004667 29070659 PMC5696646

[B57] Whitwell J. L. (2018). Tau imaging in parkinsonism: what have we learned so far? Mov. Disord. Clin. Pract. 5, 118–130. 10.1002/mdc3.12584 30035155 PMC6053061

[B58] Whitwell J. L. Lowe V. J. Tosakulwong N. Weigand S. D. Senjem M. L. Schwarz C. G.. (2017). [(18) F]AV-1451 tau positron emission tomography in progressive supranuclear palsy. Mov. Disord. 32, 124–133. 10.1002/mds.26834 27787958 PMC5552410

[B59] Wooten D. W. Guehl N. J. Verwer E. E. Shoup T. M. Yokell D. L. Zubcevik N.. (2017). Pharmacokinetic evaluation of the Tau PET Radiotracer 18F-T807 (18F-AV-1451) in human subjects. J. Nucl. Med. 58, 484–491. 10.2967/jnumed.115.170910 27660144 PMC5334185

