# Supplementary material for: Integrated 18F-T807 Tau PET, Structural MRI, and Plasma Tau in Tauopathy Neurodegenerative Disorders
Source: Front Aging Neurosci. 2021 Mar 29;13:646440. doi: 10.3389/fnagi.2021.646440 (PMC8039308; doi:10.3389/fnagi.2021.646440)
Supplement: Supplementary file 1 [file Table_1.DOCX]

**Supplementary Table 1. Tau PET ^18^F-T807 SUVR without partial-volume effect correction in different disease groups.**

| SUVR | Control | PD | CBS | PSP | FTD | AD | *p* |
| --- | --- | --- | --- | --- | --- | --- | --- |
| Global | 1.05 ± 0.07 | 1.00 ± 0.07 | 1.06 ± 0.06 | 1.08 ± 0.05 | 1.04 ± 0.08 | 1.70 ± 0.23 | 0.007 |
| Frontal | 1.04 ± 0.08 | 0.92 ± 0.09 | 1.03 ± 0.09 | 1.03 ± 0.06 | 1.00 ± 0.08 | 1.69 ± 0.27 | 0.003 |
| Lat Temporal | 1.04 ± 0.09 | 1.07 ± 0.07 | 1.12 ± 0.07 | 1.12 ± 0.06 | 1.10 ± 0.10 | 1.75 ± 0.19 | 0.009 |
| Parietal | 1.04 ± 0.09 | 1.00 ± 0.08 | 1.05 ± 0.07 | 1.13 ± 0.08 | 1.04 ± 0.10 | 1.75 ± 0.28 | 0.003 |
| Occipital | 1.08 ± 0.10 | 1.07 ± 0.11 | 1.13 ± 0.07 | 1.16 ± 0.07 | 1.06 ± 0.08 | 1.48 ± 0.20 | 0.029 |
| Putamen | 1.37 ± 0.23 | 1.31 ± 0.20 | 1.41 ± 0.25 | 1.61 ± 0.50 | 1.70 ± 0.28 | 1.86 ± 0.28 | 0.027 |
| Pallidum | 1.59 ± 0.31 | 1.49 ± 0.25 | 1.78 ± 0.29 | 2.09 ± 0.39 | 2.07 ± 0.67 | 1.43 ± 0.38 | 0.016 |

PD, Parkinson’s disease; CBS, corticobasal syndrome; PSP, progressive supranuclear palsy; FTD, frontotemporal dementia; AD, Alzheimer’s disease.

Data presented as mean ± SD. *p*: Kruskal-Wallis *p* value for SUVR between groups.

**Supplementary Table 2. Linear regression of plasma tau and ^18^F-T807 SUVR in tauopathy-related disorders**

| **Simple Linear Regression** | | | | | |
| --- | --- | --- | --- | --- | --- |
|  | Coefficient (β1) | SE | r | t | *p* |
| Global | 0.143 | 0.055 | 0.44 | 2.575 | 0.016* |
| Frontal | 0.170 | 0.058 | 0.49 | 2.938 | 0.007** |
| Lateral temporal | 0.118 | 0.057 | 0.37 | 2.050 | 0.050 |
| Parietal | 0.158 | 0.061 | 0.44 | 2.579 | 0.016* |
| Occipital | 0.077 | 0.038 | 0.36 | 2.020 | 0.053 |
| Putamen | 0.061 | 0.071 | 0.16 | 0.850 | 0.403 |
| Pallidum | 0.032 | 0.094 | 0.07 | 0.344 | 0.733 |
|  | | | | | |
| **Multiple Linear Regression: plasma tau, age, sex as covariates** | | | | | |
|  | Coefficient (β1) | SE | r (partial) | t | *p* |
| Global | 0.129 | 0.061 | 0.39 | 2.124 | 0.044* |
| Frontal | 0.152 | 0.063 | 0.43 | 2.404 | 0.024* |
| Lateral temporal | 0.104 | 0.063 | 0.31 | 1.656 | 0.305 |
| Parietal | 0.146 | 0.068 | 0.40 | 2.169 | 0.040* |
| Occipital | 0.077 | 0.042 | 0.34 | 1.834 | 0.079 |
| Putamen | 0.003 | 0.070 | 0.01 | 0.041 | 0.968 |
| Pallidum | 0.022 | 0.102 | 0.04 | 0.214 | 0.832 |

Data presented as mean ± SD.

Significant positive associations were found in global, frontal, and parietal regions in simple regression analysis, and were all still significantly associated in the multiple regression model when plasma tau, age, and sex were set as independent covariates.

*r*: correlation coefficient based on linear regression model; t: t value for the regression coefficient (β1).

**p* < 0.05; ***p* < 0.01.

**Supplementary Table 3. Correlation of ^18^F-T807 SUVR and cortical thickness in each atlas-defined regions among all patients.**

| **Region** | **Left** | | | **Right** | | | |
| --- | --- | --- | --- | --- | --- | --- | --- |
|  | ***r*** | **95% CI** | ***p*** | | ***r*** | **95% CI** | ***p*** |
| bankssts | -0.504 | -0.703 to -0.232 | <.001 | | -0.516 | -0.720 to -0.231 | .001 |
| caudalanteriorcingulate | -0.434 | -0.665 to -0.128 | .007 | | -0.469 | -0.689 to -0.171 | .003 |
| caudalmiddlefrontal | -0.309 | -0.575 to 0.017 | .063 | | -0.411 | -0.649 to -0.101 | .011 |
| cuneus | 0.194 | -0.138 to 0.488 | .249 | | 0.167 | -0.165 to 0.466 | .321 |
| entorhinal | -0.394 | -0.637 to -0.080 | .016 | | 0.043 | -0.285 to 0.362 | .799 |
| fusiform | -0.518 | -0.721 to -0.233 | .001 | | -0.449 | -0.675 to -0.146 | .005 |
| inferiorparietal | -0.461 | -0.683 to -0.161 | .004 | | -0.394 | -0.637 to -0.080 | .016 |
| inferiortemporal | -0.520 | -0.723 to -0.236 | .001 | | -0.327 | -0.588 to -0.003 | .049 |
| isthmuscingulate | -0.351 | -0.606 to -0.031 | .033 | | -0.246 | -0.528 to -0.085 | .142 |
| lateraloccipital | -0.134 | -0.439 to 0.199 | .430 | | -0.148 | -0.450 to 0.185 | .384 |
| lateralorbitofrontal | -0.394 | -0.637 to 0.080 | .016 | | -0.349 | -0.605 to -0.028 | .034 |
| lingual | -0.071 | -0.386 to 0.259 | .678 | | 0.156 | -0.177 to 0.457 | .357 |
| medialorbitofrontal | -0.245 | -0.527 to 0.086 | .144 | | -0.168 | -0.467 to -0.165 | .320 |
| middletemporal | -0.491 | -0.703 to -0.199 | .002 | | -0.277 | -0.551 to 0.052 | .098 |
| parahippocampal | -0.338 | -0.596 to -0.015 | .041 | | -0.162 | -0.462 to 0.171 | .339 |
| paracentral | -0.305 | -0.573 to 0.021 | .066 | | -0.126 | -0.432 to 0.207 | .459 |
| parsopercularis | -0.530 | -0.729 to -0.249 | <.001 | | -0.463 | -0.684 to -0.163 | .004 |
| parsorbitalis | 0.207 | -0.120 to 0.502 | .207 | | -0.011 | -0.334 to -.314 | .949 |
| parstriangularis | -0.393 | -0.636 to -0.079 | .016 | | -0.249 | -0.530 to 0.081 | .137 |
| pericalcarine | -0.001 | -0.325 to 0.323 | .993 | | 0.296 | -0.031 to 0.566 | .074 |
| postcentral | -0.298 | -0.567 to 0.029 | .073 | | -0.362 | -0.614 to -0.043 | .028 |
| posteriorcingulate | -0.623 | -0.788 to -0.375 | <.0001 | | -0.623 | -0.788 to -0.374 | <.0001 |
| precentral | -0.414 | -0.650 to -0.103 | .011 | | -0.360 | -0.613 to -0.041 | .029 |
| precuneus | -0.469 | -0.689 to -0.171 | .003 | | -0.425 | -0.658 to -0.117 | .009 |
| rostralanteriorcingulate | -0.354 | -0.608 to -0.033 | .032 | | -0.451 | -0.676 to -0.149 | .005 |
| rostralmiddlefrontal | -0.395 | -0.637 to -0.081 | .016 | | -0.107 | -0.417 to 0.225 | .529 |
| superiorfrontal | -0.412 | -0.649 to -0.102 | .011 | | -0.365 | -0.616 to -0.046 | .027 |
| superiorparietal | -0.289 | -0.560 to 0.039 | .083 | | -0.161 | -0.461 to 0.172 | .341 |
| superiortemporal | -0.165 | -0.464 to 0.168 | .330 | | -0.061 | -0.377 to 0.269 | .721 |
| supramarginal | -0.565 | -0.752 to -0.295 | <.001 | | -0.565 | -0.752 to -0.295 | <.001 |
| frontalpole | 0.314 | -0.011 to 0.579 | .058 | | 0.353 | 0.032 to 0.607 | .032 |
| temporalpole | -0.089 | -0.401 to 0.242 | .602 | | -0.034 | -0.354 to 0.293 | .841 |
| transversetemporal | -0.108 | -0.418 to 0.224 | .524 | | -0.149 | -0.451 to 0.184 | .379 |
| insula | -0.391 | -0.635 to -0.077 | .017 | | -0.296 | -0.566 to 0.031 | .008 |

Data presented as mean ± SD.

*r*: correlation coefficient. *p*: *p* value for Pearson’s correlation coefficient.
