# Supplementary figures and images for: Integrated 18F-T807 Tau PET, Structural MRI, and Plasma Tau in Tauopathy Neurodegenerative Disorders
Source: Front Aging Neurosci. 2021 Mar 29;13:646440. doi: 10.3389/fnagi.2021.646440 (PMC8039308; doi:10.3389/fnagi.2021.646440)

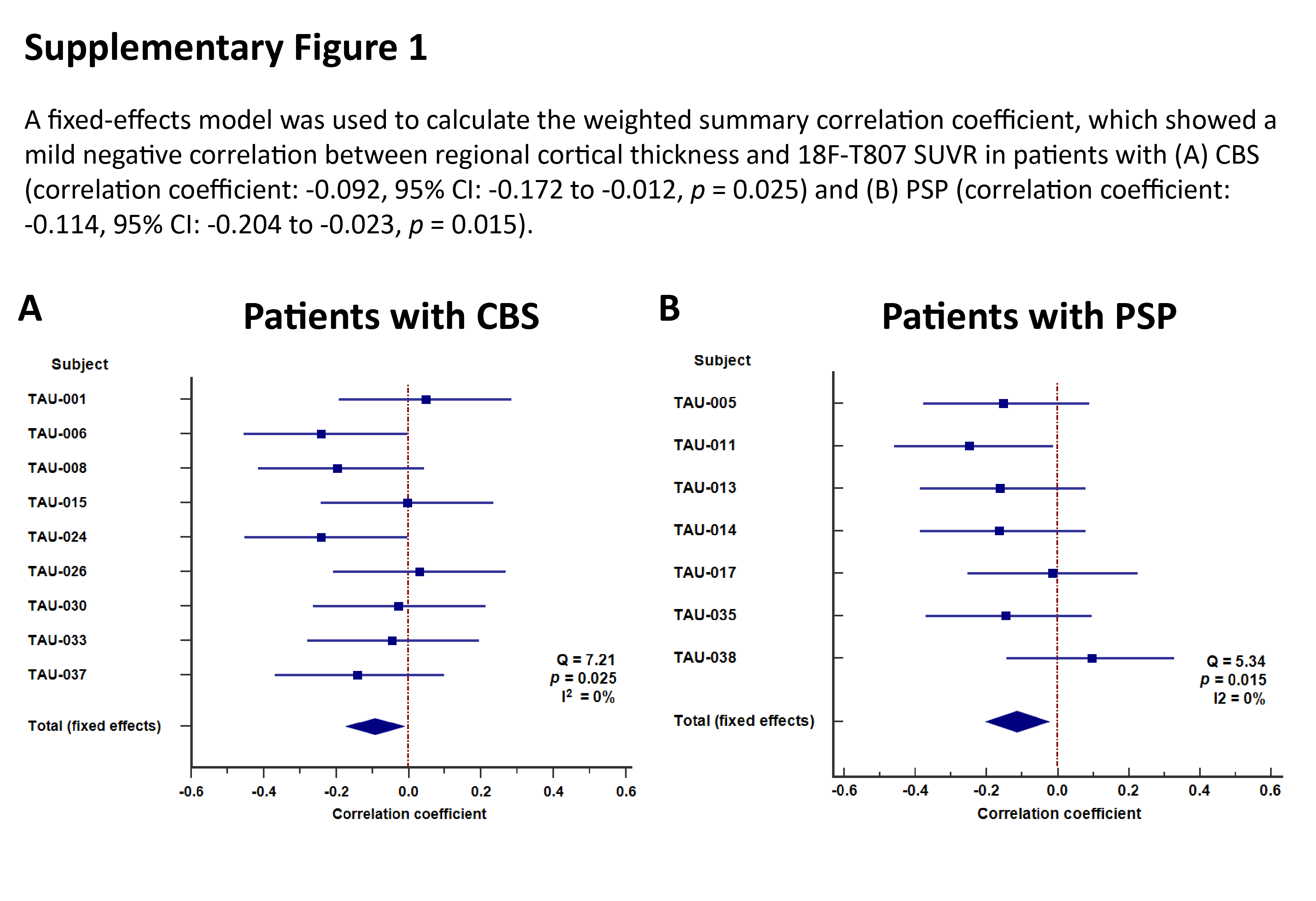

Supplement: Supplementary file 2 [file Image_1.TIF]

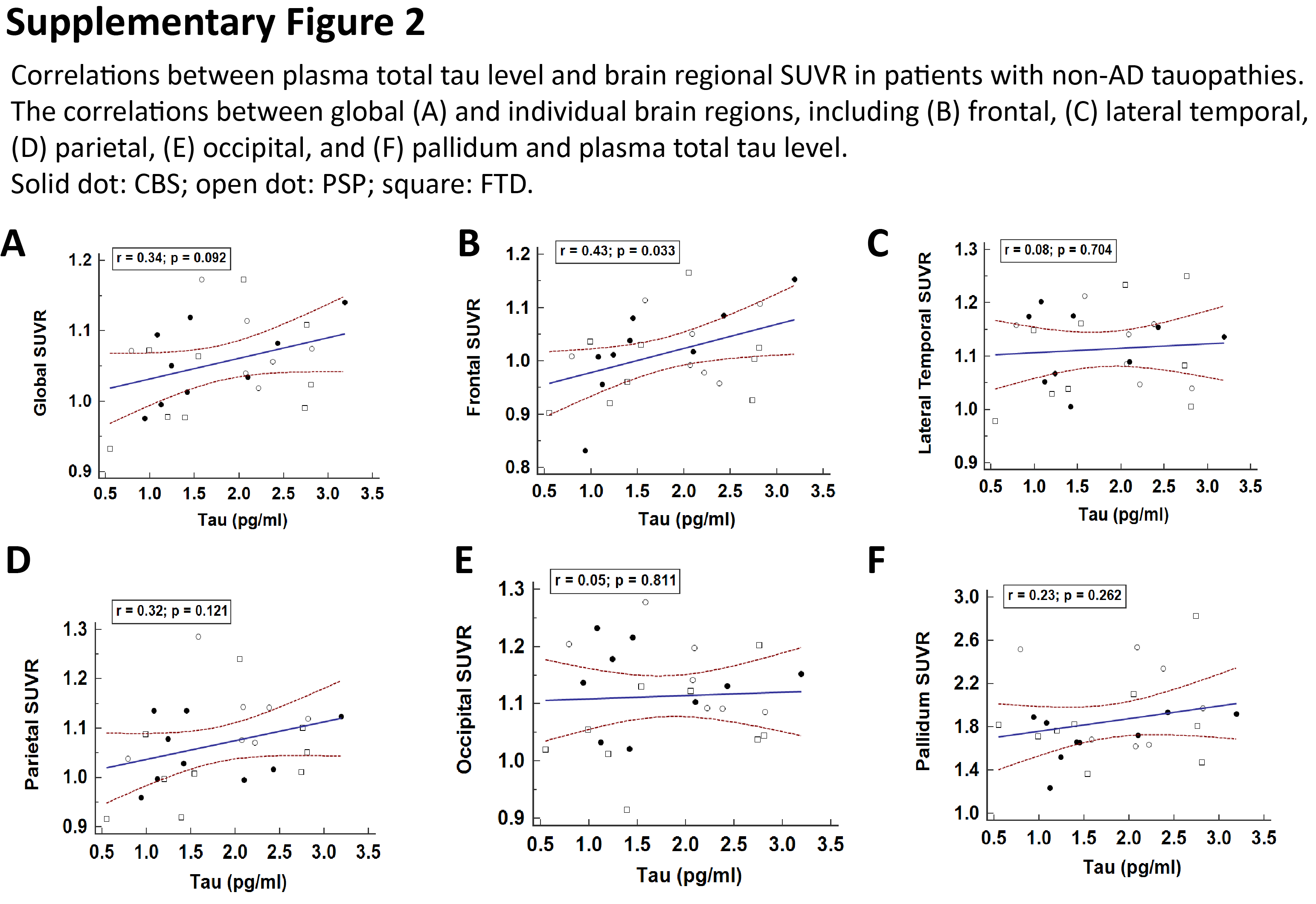

Supplement: Supplementary file 3 [file Image_2.TIF]
